# Efficiency of* Sophora flavescens*-Fructus Ligustri Lucidi Drug Pairs in the Treatment of Liver Fibrosis Based on the Response Surface Method

**DOI:** 10.1155/2019/8609490

**Published:** 2019-04-01

**Authors:** Shuhan Chu, Hongxu Zhang, Lixin Ding

**Affiliations:** Key Lab of Biological Medical Preparation, Pharmaceutical School of Jiamusi University, Jiamusi, Heilongjiang 154007, China

## Abstract

The pairing of* Sophora flavescens* and Fructus Ligustri lucidi is taken from Shi Jinmo Medicine. The idea behind this pairing was inspired by the similarity in pharmacological effects of the two herbal drugs, both of which are known to be effective in the treatment and protection against liver fibrosis. To quantitatively study the extent of the interaction between these drugs and the effect of pairing on the treatment of liver fibrosis, an animal model of liver fibrosis mice was established by intraperitoneal injection of low-dose carbon tetrachloride. The drugs were then administered individually, or in predefined compatibility ratio pairs, by gavage, and the effects on indexes of liver fibrosis were observed. The multisynthetic index method was adopted using Matlab software in order to construct a three-dimensional response surface map of the integration effect and conduct interaction analysis of* Sophora flavescens* and Fructus Ligustri lucidi. The quadratic surface fitting pattern was designed by quadratic regression to determine the optimal range of each drug. The obtained results show that when the compatibility ratio of* Sophora flavescens*-Fructus Ligustri lucidi drug pairs is less than or equal to 1:1, their therapeutic effect is enhanced by synergy (interaction value ranging between -0.2 and -1). Overall, the synergy of the high-dose drug pairs is stronger than that of the low-dose drug pairs. The optimal dose ranges are 6~12 g and 8~17 g for* Sophora flavescens* and Fructus Ligustri lucidi, respectively.

## 1. Introduction

A drug pair is a relatively fixed combination of two compatible medicines that interact synergistically in the treatment of particular ailments and infections. Such pairing of drugs, especially herbal drugs, is a technique that has been commonly used in traditional Chinese medicine (TCM), and it is the basic form of compatibility of TCM. Combining drugs alters the pharmacological and toxicological effects of the individual components, which may enhance their potency by synergistic effect. The interactions between the multiple natural components comprising a TCM are complex, and, thus, its mechanism of action is more complicated than that of single active component chemical drugs [1–5].

Hepatic fibrosis refers to the proliferation of connective tissue in the liver caused by varieties in that areas. It is a collection of various types of chronic liver diseases that lead to liver pathogenic factors, leading to pathological excessive deposition of diffuse extracellular matrix in those areas. It is a collection of various types of chronic liver diseases that lead to liver cirrhosis [6, 7]. When liver fibrosis develops into cirrhosis, normal liver function is severely impaired. However, according to the literature, liver fibrosis is reversible, which allows for the possibility of intervention and treatment by early drugs [8–10]. Therefore, it is necessary to find effective drugs to prevent liver tissue damage and treat liver fibrosis. At present, studies have shown that many TCMs have a significant effect on the treatment of liver fibrosis, as well as very good clinical application prospects [4, 5, 11]. Liver fibrosis may be chemically induced in animals using hepatotoxic agents, such as paracetamol, galactosamine, thioacetamide, and carbon tetrachloride (CCl4). The most commonly used agent is CCl4, as it processes the greatest potency for causing liver damage and fibrosis in animals. Moreover, it is stable and easy to manipulate. Induced liver fibrosis in animals is very similar to that observed in humans in terms of morphology, cellular biochemistry, and molecular changes [12]. When carbon tetrachloride (CCl4) enters the hepatic microsomes of the mouse, it affects the metabolism of hepatic cytochrome P450-dependent mixed functional oxidase in the endoplasmic reticulum of hepatocytes. This leads to the production of trichloromethyl radicals and chlorine radicals, which ultimately results in an increase in hepatocytes. In terms of molecular binding, CCl4 attacks unsaturated lipid membranes, thus, producing free radicals of reactive oxygen and triggering lipid peroxidation, which leads to liver damage, deformation, and necrosis. Long-term exposure to carbon tetrachloride in mice eventually leads to liver fiber generation [13–15]. Larger concentrations of carbon tetrachloride can immediately lead to liver damage, but the mortality risk would be high.

The drug pair of* Sophora flavescens*-Fructus Ligustri lucidi is based on description in the work* Shi Jinmo Medicine*. It is commonly used in clinical settings to protect against or to treat liver fibrosis.* Sophora flavescens* is a dry root that has anti-inflammatory, antifibrosis, antiliver cancer, antitumor, and other pharmacological effects [16, 17]. Meanwhile, Fructus Ligustri lucidi is a dry ripe fruit of the genus Oleaceae that nourishes and protects the liver and kidneys [18].* Sophora flavescens* suppresses the immunity, whereas Fructus Ligustri lucidi enhances it. The combination of the two herbs shows good therapeutic effect on various cancers.

The response surface method (RSM) is mostly used to optimize the extraction process of active ingredients of TCM [19, 20]. Recently, it has also been used to qualitatively and quantitatively investigate the pharmacodynamic interactions between drugs, as well as find the optimal threshold response range between drugs, leading to the variations in proportions and doses. Thus, the quantitative analysis of pharmacodynamic reactions/effects in drugs is more important than qualitative analysis.

Regarding best therapeutic effect and minimal adverse reactions [21–24], the study of pharmacodynamic interactions plays a very important role in guiding clinical medication and quantitative research can reflect the interaction between drugs more intuitively than other kinds of research [14, 15]. The drug interactions in TCMs are more complex due to variations in proportions and doses. Thus, the quantitative analysis of pharmacodynamic reactions/effects in drugs is more important than qualitative analysis.

In this study, we investigate the dose-effective relationship between* Sophora flavescens* and Fructus Ligustri lucidi, the two components of an herbal drug pair commonly used in TCM to treat hepatic fibrosis. Due to the complexity of the interactions between these two components, a single indicator cannot indicate the changes in the law of effectiveness. Therefore, in order to comprehensively evaluate the efficacy of the drug, various indicators of liver fibrosis were standardized and associated with different weight coefficients. By using the multi-indicator effective evaluation method, a suitable mathematical model was established. The response surface method was then used for a better indication of drug pairing interactions and the evaluation of the optimal range of effectiveness.

According to the literature, the active ingredients and pharmacodynamics of the two single drugs were studied separately. At present, there are no reports of the pair of* Sophora flavescens*-Fructus Ligustri lucidi [1, 2, 25–27].

**Figure 1:**
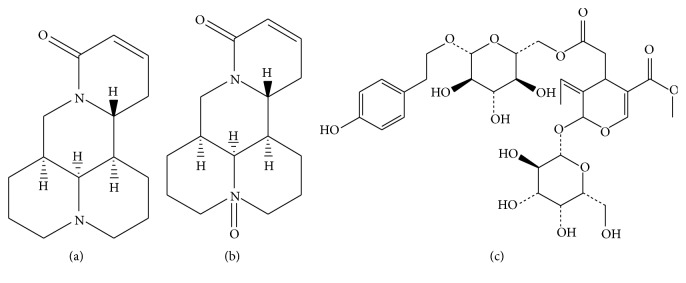
Chemical structures of (a) matrine, (b) oxymatrine, and (c) specnuezhenide.

Different proportions of* Sophora flavescens*-Fructus Ligustri lucidi have been studied for their effects on the concentration of matrine, oxymatrine, and specnuezhenide, and the pharmacological effects of the drug pairs have been studied further.  Figure 1 shows the chemical structure of the three active ingredients. Here, for the first time, the modern analysis method was used to quantitatively analyze the change of the compatibility effect of* Sophora flavescens*-Fructus Ligustri lucidi by integrating indicators with a multi-index composite index method with analysis of integration results using response surface analysis to establish the law of the interaction of* Sophora flavescens* and Fructus Ligustri lucidi on the treatment of liver fibrosis and the optimal range of drug use. The rationality and scientificity of the compatibility of* Sophora flavescens* and Fructus Ligustri lucidi are discussed here from the perspective of treatment of liver fibrosis and liver protection, which provides a scientific basis for the clinical assessment of the rational compatibility of* Sophora flavescens* and Fructus Ligustri lucidi.

## 2. Materials and Methods

### 2.1. Materials and Reagents

Both* Sophora flavescens* and Fructus Ligustri lucidi were purchased from Jintian Pharmacy of Jiamusi City. They were identified by Zong Ximing, the senior pharmacist of the Pharmacy Experimental Center at Jiamusi University School of Pharmacy, as dry roots of the genus Eucalyptus and dried fruits of the genus Oleaceae, respectively.

Alkaline Phosphatase (ALP) Assay Kit, A059-2; Total Superoxide Dismutase (SOD) Assay Kit, A001-3; A001-3, Aspartate Aminotransferase (AST), C010-2, Alanine Aminotransferase (ALT) test kit, malondialdehyde (MDA) assay kit, A003-1, and total protein (TP) assay kit, A045-2, were purchased from Nanjing Institute of Bioengineering. Colchicine was supplied by Xishuangbanna Pharmaceutical Co. Ltd., peanut oil by Shandong Luhua Group Co. Ltd., saline by Harbin Sanlian Pharmaceutical Co. Ltd., and carbon tetrachloride by analytical grade from Tianjin Kaitong Chemical Reagent Co. Ltd. Water was purified.

Meanwhile, the instruments TDZ4-WS Centrifuge, Sunrise Microplate Reader, OLYMPUS Optical Microscope, Embedding Machine, and Finesse 325 paraffin slicer, were supplied by Shanghai Luxiangyi Co. Ltd., Shanghai, In-Tech Co. Ltd., BX-41, Japan, OLYMPUS, Shenyang Yude, Electronic Instrument Co. Ltd., and Taiwei Technology Co. Ltd., respectively.

### 2.2. Preparation of Sophora Flavescens and Fructus Ligustri Lucidi Lucidum on Water Decoction

A set of seven* Sophora flavescens*-Fructus Ligustri lucidi drug pairs were prepared at ratios of 1:0, 0:1, 1:1, 1:2, 2:1, 1:3 (m:m), and 3:1. Three subsets of each set were prepared to achieve high, medium, and low dosage of single or combined drug. The different dosages were prepared by varying the amount of* Sophora flavescens* between 4.5 and 9.0 g, and that of Fructus Ligustri lucidi was between 6.0 and 12.0 g, based on the Chinese Pharmacopoeia. Thus, the high, medium, and low doses of the 1:0 drug pair were set at 9.0 g, 7.0 g, and 4.0 g, respectively, and those of the 0:1 pair were set at 12.0 g, 10.0 g, and 6.0 g, respectively. Upon weighing and mixing the appropriate amounts of the drugs for every pair, the mixture was soaked in distilled water for 1 hour and then boiled and simmered for 30 minutes. The amount of water used for each pair was determined such that it is four times the amount of the drug mixture. After bench cooling, the aqueous solution containing the drugs was collected by filtration. The process was repeated twice to ensure complete extraction, and the combined extract was then evaporated under vacuum, to maximize the drug concentrations. The samples were then stored in a refrigerator for further analysis.

### 2.3. Establishment of Animal Grouping, Liver Fibrosis Model, and Drug Delivery

Clean male KM mice weighing somewhere between 18 and 22 g were provided by the Animal Center of Harbin Medical University, with certificate number SCXK2013-001. The mice were numbered and randomly divided into 24 groups of 10 animals each. The groups were the normal group, model group, and western medicine group, as well as 21 Chinese medicine groups, each of which was treated by one of the seven sets of drug pairs at low, medium, or high dosage, prepared in this study. All of the groups were given the same free diet and drinking water. To induce liver fibrosis, all of the groups, except for the normal group, were intraperitoneally injected with 0.5% CCl4 solution prepared by dissolving 0.75 ml of CCl4, analytical pure grade, in 149.25 ml of peanut oil, at a dosage of 10*μ*l/g, once every 2 days for 6 weeks. The normal groups were intraperitoneally injected with peanut oil solution.

According to the human and animal body surface area conversion scale and the mice used (mouse dosage = human clinical dose × 0.0026/0.02 mg/kg), each group within the model was gavaged according to the corresponding drug concentration, 10*μ*l/g, 1 time/day for 6 weeks. The normal group and the western medicine group were all given the same amount of normal saline under the same conditions.

### 2.4. Material and Indicator Detection

After the last administration, the rats were fasted for 8 h. Blood was taken off the eyeball and the serum was centrifuged at 2000 r/min for 15 min. The serum was than separated and the ALT, AST, and ALP activities were detected by colorimetric method according to the kit instructions. The mice were dissected and the livers were removed and weighed. The liver index (HI) was calculated using the formula: HI = liver wet weight (mg)/mouse weight (mg). The same part of the left lobe of each liver was taken and centrifuged at 2000 r/min for 10 min in 10% liver homogenate. The supernatant was collected and the contents of SOD and MDA were measured by colorimetric method according to the kit instructions.

### 2.5. Liver Pathology Test

The liver of the mouse was rinsed to colorless, blotted dry with a filter paper and then weighed. Equal size portions of the tissue block were cut from the right lobe of the liver and fixed in 10% neutral formaldehyde solution. The tissue block was then dehydrated, waxed, sliced, and stained by HE. Finally, the pathological changes of liver tissue were observed under light microscope.

### 2.6. Statistical Analysis of Experimental Data

Statistical analysis was performed using SPSS11.0 statistical analysis software. The experimental results were expressed as x±s, using one-way variance. Analysis of variance was performed in order to compare between the different groups. The orderly qualitative data were compared by groups using nonparametric test methods. The difference was statistically significant with P < 0.05.

### 2.7. Integration Effect of Multiple Indicators

According to the multi-index comprehensive method, effective integration of multiple indicators was detected. Each indicator data was subjected to singulation; i.e., standardization = (administration group - model group) / (normal group - model group). In the past decade, 128 articles related to liver fibrosis were examined, in which the AST and ALT detected were 82 followed by 64 MDA, 62 SOD, 30 HI, and 24 ALP. The peer experts also examined the correlation between various indicators and liver fibrosis. In reference to that, the indicators had been compared to the frequency statistics reported in the literature on liver fibrosis in the past decade. The weight coefficient of each index was given independently and the expert score was determined. The coefficient of each index was determined as follows: AST, ALT is 3, MDA, SOD is 2, HI, ALP is 1. The total effect value (TE) of the therapeutic efficiency of the drug on liver fibrosis in mice was obtained by multiplying the normalized value of each indicator data by the additive value of each weight coefficient.

### 2.8. Establishment of the Pharmacodynamics Mathematical Model of Sophora flavescens-Ligustrum lucidum and the Response Surface of the Drug to the Interaction Range

After integrating the values of various indicators by the multi-index comprehensive index method, the nonlinear regression method was used to determine the parameters of the dose-response curve. The response surface model and the map of the integration effect of Sophora flavescens and Fructus* Ligustrum lucidum* were established in 3D and constructed by Matlab software. Interaction analysis was also carried out, and the quadratic surface fitting pattern was obtained by quadratic regression design. Ultimately, the optimal compatibility range of every drug pair was determined. In order to simplify the drawing and improve comprehensibility, the composite index is reduced by 1, with 0 as a typical additive.

## 3. Results and Discussion

### 3.1. Appearance and Pathological Observation of Liver Cell Damage

**Figure 2:**
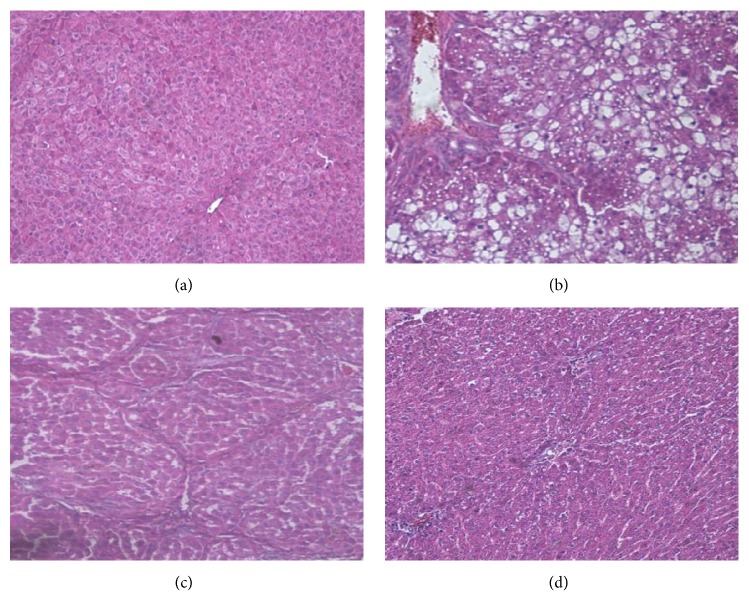
Pathological section of mouse liver tissue (×400), (a) control group; (b) model group; (c) positive control group; and (d) Chinese medicine group (1:2) (3.58 cm×5.00 cm).

The mice in the normal group were agile with shiny and dense coats, and they had a regular diet and were drinking water. Their livers had a rosy appearance, smooth surface, and soft texture, with no adhesion to surrounding tissues. A microscope examination (Figure 2) showed that the liver tissue of the normal group mice was clear, with uniform hepatocyte size, and there were no signs of degeneration or necrosis. The structure of the hepatic lobule was well-defined, and the hepatocyte cords in the lobules were arranged neatly.

Liver fibrosis mice showed relatively slow movements, mental dysfunction, yellow and sparse hair, and loss of appetite. The surfaces of their livers appeared to be slightly rough in texture. Under microscopic observation (Figure 2), the normal structure of the liver tissue was found to be destroyed, and the hepatic cord arrangement was disordered. A proliferation of fibrous tissue was observed, along with a disappearance of the lobular structure of the liver, and the appearance of balloon-like cells, indicative of steatosis. Finally, a large number of inflammatory cells were seen around the portal area and the central vein. The infiltration observation showed a significant difference between the liver fibrosis model group and the normal group, with P < 0.05.

After single or combined drug treatment, the liver state was significantly improved. The extent of therapeutic effectiveness depended on the compatibility ratio of the drug pair. However, none of the seven investigated treatment sets could fully restore the liver to its normal state (Figure 2).

### 3.2. Changes in Various Indicators and Liver Index

As can be seen from Table 1, compared to the normal group, the ALT, AST, MDA, ALP, and HI levels in the model group are significantly increased (P < 0.01), whereas the SOD level is greatly decreased (P < 0.01), indicating that liver fibrosis in the model group of mice was successfully achieved. A comparison of the results obtained for the* Sophora flavescens* single-drug treatment groups and those of the model group shows that the low-dose treatment had no significant effect on various indexes and, thus, has minimal therapeutic potency. The medium dose treatment significantly increased the SOD level (P < 0.05) and decreased the MDA level (P < 0.05), but it did not have any effect on other indicators. The group treated with high-dose* Sophora flavescens* presented a decrease in AST, MDA, HI, and ALP levels (P < 0.01), as well as a more potent decrease in SOD levels (P < 0.05). However, there was no significant improvement pertaining to the ALP index. The results indicate that only the high-dose* Sophora flavescens* treatment is effective in curing liver fibrosis in mice.

The therapeutic effectiveness of Fructus Ligustri lucidi was also evaluated by studying the influence of the low, medium, and high dosage single-drug treatments on the various indicators, as compared to the model group. The comparison shows that the low-dose treatment increased the SOD level (P < 0.01) and decreased the HI level (P < 0.01), but it had no significant effect on the other indicators. Meanwhile, the medium dose treatment only affected the levels of SOD, AST, and HI (P < 0.05), significantly. Finally, the high-dose treatment had a considerable effect on decreasing the levels of AST, MDA, HLP, and HI (P < 0.05), and increasing the levels of SOD levels (P < 0.01). Similar to* Sophora flavescens* single-drug treatment, no major improvement in the ALP index was observed for Fructus Ligustri lucidi, even in the case of the high-dose treatment. Again, the results show that, among the three investigated subsets, only the high-dose treatment exhibits appreciable therapeutic potency.

All of the investigated subsets, low, medium, and high-dose, of the* Sophora flavescens*-Fructus Ligustri lucidi drug pair showed significantly decreased levels of ALT, AST, MDA, ALP, and HI (P < 0.05) compared to the model group, except for the 3:1 subsets that did not have a significant effect on ALT levels. Meanwhile, these groups, including the 3:1 ratio groups, all exhibited a considerable increase in SOD levels (P < 0.05).

To summarize,* Sophora flavescens* and Fructus Ligustri lucidi are both effective in treating liver fibrosis as single drugs, but only at high dosage. However, the therapeutic effect is substantially improved when the drugs are paired, which is indicative of synergistic behavior.

### 3.3. Standardized Values and Integration Effects of Various Indicators

The multi-index comprehensive index method was used to standardize and integrate the indicators and liver indexes. The results are shown in Table 2. In the Chinese Pharmacopoeia, within the scope of the two single drugs, the therapeutic effects of* Sophora flavescens* and Fructus Ligustri lucidi on liver fibrosis mice were positively correlated with the dose; i.e., the higher the drug dose, the more effective the treatment. This trend is maintained for all of the investigated drug pairs, at different ratios and dosages, except for the 1:1 drug pairs. Surprisingly, the 1:1 drug pairs exhibit higher potency at low and medium doses than at high doses. The most effective drug pair was found to be the 1:2 high-dose* Sophora flavescens*-Fructus Ligustri lucidi pair, whereas the 3:1 low-dose pair showed the least effectiveness, medium dose; the other 4 ratios were treated with high-dose > medium dose > low-dose. Among the ratios of* Sophora flavescens* and Fructus Ligustri lucidi, the 1:2 high-dose group had the best treatment effect, and the 3:1 low-dose group had the worst treatment effect.

### 3.4. Evaluation of the Interaction between Sophora flavescens-Fructus Ligustri Lucidi and the Determination of the Optimal Drug Use Range

**Figure 3:**
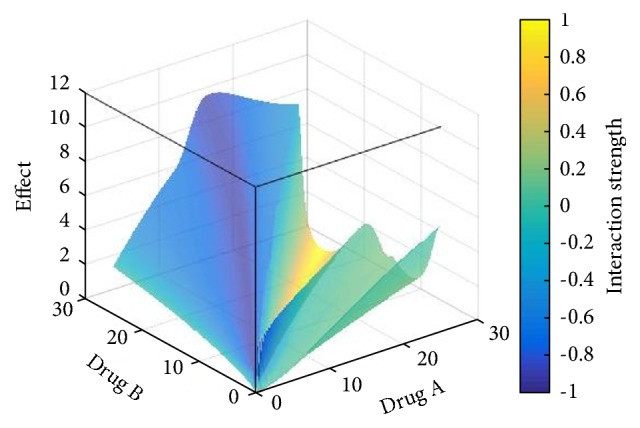
Three-dimensional response surface diagram of* Sophora flavescens*-Fructus Ligustri lucidi combination (Drug A is* Sophora flavescens*; Drug B is Fructus Ligustri lucidi. The abscissa is the dose, g).

In the three-dimensional response surface diagram presented in Figure 3, the depth of the color represents the strength of the interaction between the two drugs. If the calculated value of the comprehensive index is close to 0, then the drugs are additive, and their interaction is represented by the green color. Meanwhile, a comprehensive index value close to 1 is indicative of an antagonistic effect between the drugs, which is represented by the yellow color. An index close to -1 signifies a synergistic relationship between the drugs and is represented by the blue color. The higher the index value, the greater the intensity of the interaction and the depth of the color in the diagram.

**Figure 4:**
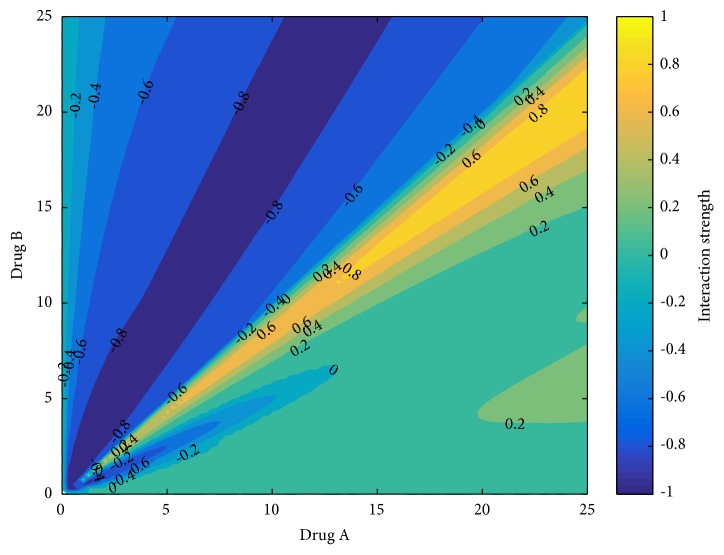
Sectional response surface diagram of* Sophora flavescens*-Fructus Ligustri lucidi combination (Drug A is* Sophora flavescens*; Drug B is Fructus Ligustri lucidi. The abscissa is the dose, g).

As shown in Figure 3, which depicts the response surface, the dug pair of* Sophora flavescens*-Fructus Ligustri lucidi has a synergistic effect on more than half of the regions. As shown in Figure 4 that when bitterness participates in the dose compatibility of Fructus Ligustri lucidi <1:1, it has a synergistic effect (the interaction value is 0 to -1), especially when the ratio of* Sophora flavescens*-Fructus Ligustri lucidi is 1:2, the treatment showed a strong synergistic effect (the part with an interaction value of -1), and, regarding the overall effect of the drug pair, the synergy observed in the high-dose group was more pronounced than that of in the low-dose group.

**Figure 5:**
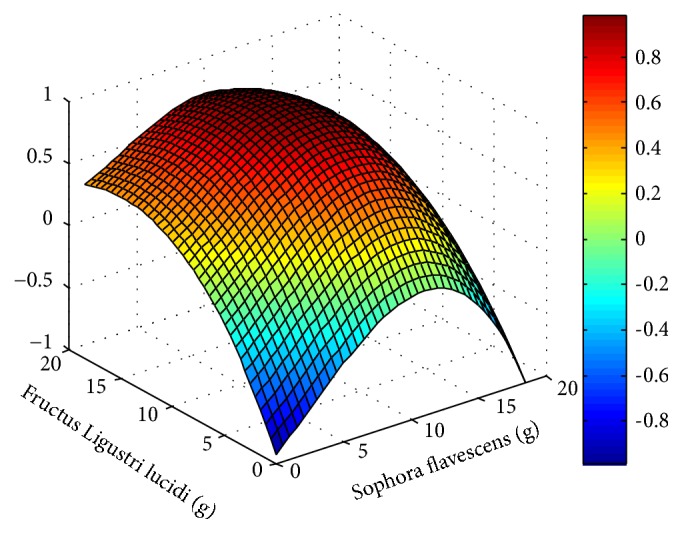
Response surface map of* Sophora flavescens*-Fructus Ligustri lucidi compatibility dose ranges.

**Figure 6:**
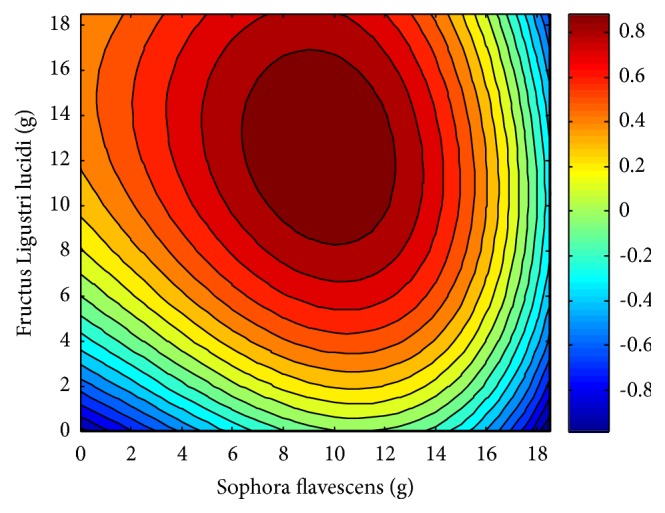
Contour map of* Sophora flavescens*-Fructus Ligustri lucidi compatibility dose ranges.

The quadratic surface fitting design, determined using Matlab software, is shown in Figure 5. This design is used to evaluate the compatibility dose ranges of the investigated drug pairs. The ranges characterized by a deep red color exhibit the highest efficacy of the* Sophora flavescens*-Fructus Ligustri lucidi drug pair. As can be seen from the contour diagram depicted in Figure 6, when the two herbal drugs are combined together, the best dosage range is 6~12 g for* Sophora flavescens* and 8~17 g for Fructus Ligustri lucidi.

### 3.5. Discussion

In this study, blood liver related indicators were used to examine the therapeutic effects of* Sophora flavescens* and Fructus Ligustri lucidi on liver fibrosis in mice. The disease was induced via intraperitoneal injections of carbon tetrachloride in peanut oil solution. It was found that these injections decreased the levels of ALT, AST, MDA, and ALP, while significantly increasing SOD levels. The liver index (HI) was appreciably decreased, indicating that liver fibrosis was successfully established.

In the western medicine group of mice, the positive control group, liver fibrosis was treated using colchicine, a chemically synthesized medicine that is known to inhibit the deposition of collagen tissue in liver tissue caused by CCl_4_ [28]. Colchicine also improves impaired liver function and is effective in preventing CCl4-induced liver fibrosis. Many studies related to drug treatment of carbon tetrachloride-induced liver fibrosis have also used colchicine in positive control groups [7, 14, 15]. It is also cheap and readily available. For this reason, studies such as the present work on carbon tetrachloride-induced liver fibrosis can also use colchicine as a positive control group.

All of the other investigated groups were treated with* Sophora flavescens* and/or Fructus Ligustri lucidi, at varying combination ratios and dosages. The results showed that the pairing of the two drugs significantly enhanced their therapeutic efficiency. The analysis of the 3D response surface graph showed that the strength of the interaction between the two herbal drugs depends on the particular combination ratio and dose. For example, drug pairs wherein the content of* Sophora flavescens* is less than that of Fructus Ligustri lucidi exhibit improved therapeutic potency, due to synergistic effects. The drug combination showing the highest potency is the 1:2* Sophora flavescens*-Fructus Ligustri lucidi combination, for which the synergistic intensity is -1.

To determine the optimal dose range for each herbal drug, quadric surface fitting analysis was performed using Matlab. The results indicated that the optimal range for Sophora flavescens is 6~12 g, whereas that of Fructus Ligustri lucidi is 8~17 g. When viewed with the analysis of the degree of interaction, it can be determined that when the dosage of* Sophora flavescens* is higher than that of Fructus Ligustri lucidi within this range, this drug can exert the maximum efficacy. These ranges are very similar to those reported in* Shi Jinmo Medicine* (6~10 g for* Sophora flavescens* and 10~15 g of Fructus Ligustri lucidi).

The effect of combination ratio of the investigated drug pairs on the content of the three active components (matrine, oxymatrine, and specnuezhenide) and their dissolution was studied. There were different ratios of* Sophora flavescens*-Fructus Ligustri lucidi (1:3, 1:2, 1:1, 2:1, 3:1), when compared with the single-taste medicine of* Sophora flavescens*; the content of oxymatrine of matrine increased with the proportion of* Sophora flavescens* and the dissolution rate of matrine increased with the proportion of compatibility. However, the dissolution rate of oxymatrine increased with the compatibility ratio of 3:1, 2:1, and 1:1, and the dissolution rate peaked when the compatibility ratio was 1:2. When the compatibility ratio was 1:3, the dissolution rate decreased but only after 3:1. Compared with the single-drug medicine of Fructus Ligustri lucidi, the content of specnuezhenide increases with the increasing proportion of Fructus Ligustri lucidi. When the compatibility ratio was 3:1, 2:1, 1:1, and 1:2, the dissolution rate of the specnuezhenide increases with the decrease of the compatibility ratio. When the compatibility ratio was 1:3, the dissolution rate of specnuezhenide was second only to the compatibility ratio at 1:2. When the compatibility ratio was 3:1, 2:1, 1:1, and 1:2, the total content and dissolution rate of the three active ingredients were all the highest at 1:2 and when the compatibility ratio is 1:3, the total content and dissolution rate of the active ingredients are second only to 1:2. Therefore, when the ratio of matrine to the mixture of* Sophora flavescens*-Fructus Ligustri lucidi is <1:1, it is conducive to the dissolution of the effective components of matrine, oxymatrine, and specnuezhenide. When the ratio is 1:2, the total content and total dissolution rate of matrine, oxymatrine, and specnuezhenide reach the highest value.

Jing Minqi [29] reported that oxymatrine of matrine is the main active ingredient in* Sophora flavescens*, and it has pharmacological effects such as antitumor and antifibrotic effects. Hu Dongmei [30] reported that the specnuezhenide is a characteristic active component of Fructus Ligustri lucidi and is that it has a high level of ciliated cyclic iridoid glycosides in the Ligustrum. It has pharmacological effects such as hepatoprotective, anti-inflammatory, and antitumor effects. For this reason, this study combines the effects of different ratios of the early matrine and* Ligustrum lucidum* on the content and dissolution of three active ingredients in* Sophora flavescens* and* Ligustrum lucidum*. We here observed* Sophora flavescens*-*Ligustrum lucidum* at different ratios, showing differences in the degree and extent of synergy, antagonism. It is closely related to changes in its active ingredients.

## 4. Conclusion 

The results show that drug pairs with compatibility ratios < 1:1 exhibit improved therapeutic efficiency compared to the individual drugs, due to synergistic effects. The strongest synergy and greatest healing potency were observed for the 1:2* Sophora flavescens*-Fructus Ligustri lucidi drug pair. In general, the overall performance of the high-dose drug pairs was stronger than that of the low-dose drug pairs. Quadric surface fitting analysis, carried out using Matlab, showed that the optimal dosage range for* Sophora flavescens* is 6~12 g, whereas that of Fructus Ligustri lucidi is 8~17 g, compared to 6~10 g and 10~15 g, respectively, as reported in* Shi Jinmo Medicine*. To a certain extent, the similarity between the reported values and those calculated in this study proves the rationality of classic ancient prescriptions, and it provides a scientific basis for the clinical use of traditional Chinese medicine. This research provides insight into the science of drug pairing, and it is valuable in guiding the clinical use of* Sophora flavescens* and Fructus Ligustri lucidi herbal drugs.

## Figures and Tables

**Table 1:** Effect of *Sophora flavescens*-Fructus Ligustrum lucidum drug pair treatment on blood and liver indexes in mice with liver fibrosis (x±s, n=8), for different drug ratios (1:0, 0:1, 1:1, 1:2, 1:3, 2:1, and 3:1) and dosages (low (L), medium (M), and high (H)).

Group	ALT	AST	SOD	MDA	ALP	HI
Normal	24.81±1.90	94.38±5.46	322.40±9.29	8.03±0.14	64.96±3.36	3.39±0.17
Model	118.62±5.90##	318.28±4.33##	160.47±2.79##	24.47±0.89##	191.61±3.71##	9.77±0.01##
Positive control	38.71±2.14*∗∗*	128.38±6.25*∗∗*	299.41±7.05*∗∗*	9.11±0.45*∗∗*	72.39±3.93*∗∗*	3.81±0.7*∗∗*
1:0 (L)	116.52±7.30	312.39±2.84	163.15±4.19	23.49±0.87	189.52±2.50	9.31±0.92
1:0 (M)	113.82±5.54	309.32±10.37	172.07±1.14*∗*	22.55±1.48*∗*	187.38±4.28	9.19±1.00
1:0 (H)	110.40±6.36	304.13±8.63*∗*	176.23±6.97*∗*	22.34±1.31*∗*	181.56±9.33*∗∗*	9.09±0.37*∗*
0:1 (L)	115.75±3.71	307.14±9.84	180.22±4.15*∗∗*	23.20±0.80	189.94±1.31	9.09±0.72*∗∗*
0:1 (M)	113.34±5.27	301.29±8.96*∗*	185.80±10.32*∗∗*	23.05±1.21	185.40±5.09	8.51±0.57*∗∗*
0:1 (H)	111.92±7.45	300.04±5.25*∗*	188.18±6.41*∗∗*	21.00±0.39*∗*	181.07±4.24*∗∗*	8.69±0.84*∗∗*
1:1 (L)	105.33±3.83*∗*	224.43±7.15*∗∗*	231.12±4.24*∗∗*	17.06±0.94*∗∗*	140.19±5.09*∗∗*	6.69±0.53*∗∗*
1:1(M)	94.53±7.00*∗∗*	183.29±6.71*∗∗*	240.52±6.67*∗∗*	15.32±1.27*∗∗*	128.92±3.65*∗∗*	6.15±0.86*∗∗*
1:1 (H)	101.81±11.72*∗*	171.04±6.19*∗∗*	233.28±7.29*∗∗*	14.3±1.01*∗∗*	121.63±3.28*∗∗*	6.23±0.877*∗∗*
1:2 (L)	77.71±5.66*∗∗*	152.46±7.17*∗∗*	273.17±5.96*∗∗*	12.84±0.74*∗∗*	98.46±3.86*∗∗*	4.59±0.108*∗∗*
1:2 (M)	70.46±3.63*∗∗*	134.35±2.65*∗∗*	280.28±4.41*∗∗*	10.15±2.74*∗∗*	81.31±2.96*∗∗*	4.19±0.46*∗∗*
1:2 (H)	64.54±5.33*∗∗*	127.88±3.88*∗∗*	289.46±4.10*∗∗*	9.66±0.88*∗∗*	79.33±4.21*∗∗*	4.46±0.05*∗∗*
1:3 (L)	87.33±4.84*∗∗*	298.51±5.16*∗*	248.53±3.33*∗∗*	13.11±1.12*∗∗*	108.48±4.26*∗∗*	5.71±0.52*∗∗*
1:3(M)	84.27±6.00*∗∗*	290.53±12.22*∗∗*	260.45±9.08*∗∗*	14.01±1.52*∗∗*	113.24±5.32*∗∗*	5.36±0.51*∗∗*
1:3 (H)	82.56±3.77*∗∗*	291.87±7.7*∗∗*	270.52±7.93*∗∗*	12.88±0.13*∗∗*	101.14±3.38*∗∗*	5.27±0.14*∗∗*
2:1 (L)	105.59±7.78*∗*	272.17±11.48*∗∗*	214.52±6.00*∗∗*	18.45±0.54*∗∗*	160.97±4.26*∗∗*	6.44±0.08*∗∗*
2:1 (M)	104.54±6.91*∗*	270.52±8.42*∗∗*	213.03±4.53*∗∗*	18.75±0.26*∗∗*	157.42±3.81*∗∗*	6.63±0.60*∗∗*
2:1(H)	106.36±8.84*∗*	270.39±3.93*∗∗*	210.72±3.63*∗∗*	18.05±0.81*∗*	153.43±2.25*∗∗*	6.76±0.37*∗∗*
3:1 (L)	110.72±7.75	298.514±6.01*∗*	189.45±2.9*∗∗*	20.55±0.40*∗∗*	182.66±2.23*∗∗*	8.36±0.65*∗∗*
3:1 (M)	108.83±4.06	290.53±7.00*∗∗*	192.82±2.64*∗∗*	20.11±1.24*∗∗*	174.53±4.95*∗∗*	8.15±0.435*∗∗*
3:1 (H)	109.44±6.69	291.81±3.87*∗∗*	197.12±2.89*∗∗*	20.18±1.17*∗∗*	162.39±4.14*∗∗*	7.77±0.90*∗∗*

^##^ *P* < 0.01 versus normal group; *∗P* < 0.05 *∗∗P* < 0.01 versus model group.

**Table 2:** Standardized indexes and total effect values of the investigated *Sophora flavescens*-Fructus Ligustrum lucidum drug pairs, as well as the single drugs. L, M, and H refer to low, medium, and high dosage, respectively, and TE refers to total effect.

Group	ALT	AST	SOD	MDA	ALP	HI	TE
1:0 (L)	0.022	0.026	0.017	0.060	0.017	0.072	0.387
1:0 (M)	0.051	0.0040	0.067	0.117	0.033	0.091	0.765
1:0 (H)	0.088	0.063	0.097	0.130	0.079	0.107	1.093
0:1 (L)	0.031	0.050	0.122	0.077	0.013	0.107	0.761
0:1 (M)	0.056	0.076	0.156	0.086	0.049	0.197	1.126
0:1 (H)	0.071	0.081	0.171	0.211	0.083	0.169	1.472
1:1 (L)	0.142	0.419	0.436	0.451	0.406	0.483	4.346
1:1 (M)	0.257	0.603	0.494	0.557	0.495	0.567	5.744
1:1 (H)	0.179	0.658	0.405	0.619	0.553	0.555	5.667
1:2 (L)	0.436	0.741	0.692	0.707	0.735	0.812	7.876
1:2 (M)	0.513	0.821	0.740	0.871	0.871	0.875	8.970
1:2 (H)	0.576	0.850	0.798	0.901	0.887	0.832	9.395
1:3 (L)	0.334	0.088	0.544	0.636	0.619	0.636	4.881
1:3 (M)	0.366	0.118	0.617	0.691	0.656	0.691	5.443
1:3 (H)	0.384	0.118	0.680	0.705	0.714	0.705	5.695
2:1 (L)	0.139	0.206	0.334	0.366	0.242	0.522	3.199
2:1 (M)	0.150	0.213	0.325	0.348	0.270	0.492	3.197
2:1 (H)	0.131	0.214	0.310	0.391	0.301	0.472	3.510
3:1 (L)	0.084	0.088	0.179	0.238	0.071	0.221	1.842
3:1 (M)	0.104	0.124	0.200	0.265	0.135	0.254	2.003
3:1 (H)	0.098	0.118	0.226	0.261	0.559	0.313	2.494

## Data Availability

The data used to support the findings of this study are included within the article.

## References

[1] Li S., Lin H., Tang Y. (2015). Comparative metabolomics analysis on invigorating blood circulation for herb pair Gui-Hong by ultra-high-performance liquid chromatography coupled to quadrupole time-of-flight mass spectrometry and pattern recognition approach. *Journal of Pharmaceutical and Biomedical Analysis*.

[2] Li Q., Fan Y.-S., Gao Z.-Q., Fan K., Liu Z.-J. (2015). Effect of Fructus Ligustri Lucidi on osteoblastic like cell-line MC3T3-E1. *Journal of Ethnopharmacology*.

[3] Zeng H., Xue P., Su S. (2016). Comparative Pharmacokinetics of three major bioactive components in rats after oral administration of Typhae Pollen-Trogopterus Feces drug pair before and after compatibility. *Journal of Pharmaceutical Sciences*.

[4] Li W., Hong B., Li Q., Li Z., Bi K. (2019). An integrated serum and urinary metabonomic research of Rhizoma Curcumae-Rhizoma Sparganii drug pair in hysteromyoma rats based on UPLC-Q-TOF-MS analysis. *Journal of Ethnopharmacology*.

[5] Li M., Feng X., Deng Ba D. J. (2019). Hepatoprotection of Herpetospermum caudigerum Wall. against CCl4-induced liver fibrosis on rats. *Journal of Ethnopharmacology*.

[6] Lee U. E., Friedman S. L. (2011). Mechanisms of hepatic fibrogenesis. *Best Practice & Research: Clinical Gastroenterology*.

[7] Zhao X., Li R., Liu Y. (2017). Polydatin protects against carbon tetrachloride-induced liver fibrosis in mice. *Archives of Biochemistry and Biophysics*.

[8] Campana L., Iredale J. (2017). Regression of liver fibrosis. *Seminars in Liver Disease*.

[9] Fink S. A., Jacobson I. M. (2011). Managing patients with hepatitis-B-related or hepatitis-C-related decompensated cirrhosis. *Nature Reviews Gastroenterology & Hepatology*.

[10] Popov Y., Schuppan D. (2009). Targeting liver fibrosis: strategies for development and validation of antifibrotic therapies. *Hepatology*.

[11] Tao Y. Y., Yan X. C., Zhou T. (2014). Fuzhen Huayu recipe alleviates hepatic fibrosis via inhibiting TNF – alpha induced hepatocyte apoptosis. *BMC Complementary and Alternative Medicine*.

[12] Sokar S. S., El-Sayad M. E., Ghoneim M. E., Shebl A. M. (2017). Combination of Sitagliptin and Silymarin ameliorates liver fibrosis induced by carbon tetrachloride in rats. *Biomedicine & Pharmacotherapy*.

[13] Guo Y., Liang X., Meng M. (2017). Hepatoprotective effects of Yulangsan flavone against carbon tetrachloride (CCl4)-induced hepatic fibrosis in rats. *Phytomedicine*.

[14] Liu X., Tang C., Zheng H. (2018). Investigation of the hepatoprotective effect of Corydalis saxicola Bunting on carbon tetrachloride-induced liver fibrosis in rats by 1H-NMR-based metabonomics and network pharmacology approaches. *Journal of Pharmaceutical and Biomedical Analysis*.

[15] Liu P., Shang E., Zhu Y., Qian D., Duan J. (2018). Volatile component interaction effects on compatibility of Cyperi Rhizoma and Angelicae Sinensis Radix or Chuanxiong Rhizoma by UPLC-MS/MS and response surface analysis. *Journal of Pharmaceutical and Biomedical Analysis*.

[16] Shi L., Tang X., Dang X. (2015). Investigating herb-herb interactions: the potential attenuated toxicity mechanism of the combined use of Glycyrrhizae radix et rhizoma (Gancao) and Sophorae flavescentis radix (Kushen). *Journal of Ethnopharmacology*.

[17] Yang H., Zhou Z., He L. (2018). Hepatoprotective and inhibiting HBV effects of polysaccharides from roots of Sophora flavescens. *International Journal of Biological Macromolecules*.

[18] Seo H. L., Baek S. Y., Lee E. H. (2017). Liqustri lucidi Fructus inhibits hepatic injury and functions as an antioxidant by activation of AMP-activated protein kinase in vivo and in vitro. *Chemico-Biological Interactions*.

[19] Liu Y., Gong G., Zhang J. (2014). Response surface optimization of ultrasound-assisted enzymatic extraction polysaccharides from Lycium barbarum. *Carbohydrate Polymers*.

[20] Jang S., Lee A. Y., Lee A. R., Choi G., Kim H. K. (2017). Optimization of ultrasound-assisted extraction of glycyrrhizic acid from licorice using response surface methodology. *Integrative Medicine Research*.

[21] Short T. G., Ho T. Y., Minto C. F. (2002). Efficient trial design for eliciting a pharmacokinetic-pharmacodynamic model-based response surface describing the interation between two intravenous anesthetic drugs. *Anesthesiology*.

[22] Dahan A., Nieuwenhuijs D., Olofsen E., Sarton E., Romberg R., Teppema L. (2001). Response surface modeling of alfentanil-sevoflurane interaction on cardiorespiratory control and Bispectral Index. *Anesthesiology*.

[23] Jin Y., Qu C., Tang Y. (2016). Herb pairs containing Angelicae Sinensis Radix (Danggui): a review of bio-active constituents and compatibility effects. *Journal of Ethnopharmacology*.

[24] Bohlooli F., Sepehri S., Razzaghi-Asl N. (2017). Response surface methodology in drug design: A case study on docking analysis of a potent antifungal fluconazole. *Computational Biology and Chemistry*.

[25] Zhang Q., Yu J., Zhang L., Hu M., Xu Y., Su W. (2016). Extraction, characterization, and biological activity of polysaccharides from Sophora flavescens Ait.. *International Journal of Biological Macromolecules*.

[26] Zhou Y., Wu Y., Deng L. (2014). The alkaloid matrine of the root of Sophora flavescens prevents arrhythmogenic effect of ouabain. *Phytomedicine*.

[27] Li Y.-P., Wang S.-J., Zang Y.-M., Hu Z.-S., Liu C.-S. (2016). Sources of varieties and quality of circular Fructus Ligustri Lucidi. *Chinese Journal of Natural Medicines*.

[28] He Y. J., Shu J. C., Lv X. (2006). Observation on the prevention of liver fibrosis by colchicine. *Journal of Guangdong College of Pharmacy*.

[29] Jing M. Q., Zhan H. P., Zhao M. (2018). Study on the contraindication mechanism of matrine cucurbit counter-drug based on HPLC. *Journal of Shenyang Pharmaceutical University*.

[30] Hu D. M., Lu Y., Fang M. F. (2016). Protective effect of tetraside on acute liver injury induced by carbon tetrachloride in mice. *Chinese Pharmacological Bulletin*.

